# Correlation between proteome changes and synchrony of cardiac electrical excitation under 3-day «dry immersion» conditions

**DOI:** 10.3389/fphys.2023.1285802

**Published:** 2023-12-01

**Authors:** L. H. Pastushkova, A. G. Goncharova, V. B. Rusanov, A. M. Nosovsky, D. N. Kashirina, O. V. Popova, I. M. Larina

**Affiliations:** State Scientific Center of the Russian Federation, Institute of Medical and Biological Problems Russian Academy of Sciences, Moscow, Russia

**Keywords:** proteome, cardiovascular system (CVS), protrombin, dry immersion, calmodulin

## Introduction

Cardiac mechano-electrical feedback is the effect of cardiac muscle contraction on its electrical activity (Quinn et al., 2014). Mechano-electrical feedback is a contour of regulation of cardiac muscle function. Mechanical heterogeneity or mechanical ventricular dyssynchrony of the heart is accompanied by electrical remodeling and an increase in myocardial electrical heterogeneity (Kamkin and Iu, 2012; Jeyaraj et al., 2007). The magnitude of mechanical load on cardiac muscle significantly affects not only the nature of mechanical activity of cardiomyocytes, but also their electrical activity. The risk of heart rhythm disturbances development as a reflection of total processes of myocardial electrical excitation desynchronization in practically healthy subjects from the group of special contingent (cosmonauts, volunteer testers) makes the study of blood proteomic composition peculiarities relevant to regulation of myocardial repolarization and depolarization processes. The common prevalence of atrial fibrillation in active astronauts is ≈5%, similar to the general population but at a younger age. Risk factors for atrial fibrillation are left atrial enlargement, increased number of premature atrial complexes, and certain electrocardiogram parameters: P waveform duration, RMS voltage at the last 20 ms of the P waveform with signal averaging, and P waveform amplitud.

According to Khine H.W. et al. in thirty astronauts who flew in space missions of 6 months’ duration, transient changes in the structure of the left atrium and changes in atrial electrophysiology, which increase the risk of atrial fibrillation, were detected. However, no definite signs of increased supraventricular arrhythmias and no detectable episodes of AF were found (Khine et al., 2018).

It is known that subjects in the state of dry immersion (DI) register the same reactions as astronauts in the first week on the ISS (Tomilovskaya et al., 2018). DI contributes to the development of rapid gravitational deconditioning of the somatosensory, cardiovascular, and other systems (Navasiolava et al., 2011; Tomilovskaya et al., 2021).

It has been noted that 7-day DI causes changes in autonomic regulation and changes in myocardial state, which are manifested in the growth of sympathetic regulation activity, decrease in the functional reserve of regulatory systems, decrease in systolic blood pressure and deterioration of myocardial electrophysiological characteristics. All these shifts are signs of adaptive restructuring of regulatory mechanisms, which leads, among other things, to a decrease in orthostatic stability (Larina et al., 2011).

In the experiment with 21-day DI it was shown that model conditions sharply changed hemodynamic and baroreflex response both at rest and during special tests (Borovik et al., 2020).

However, studies of blood proteome dynamics reflecting myocardial electrical excitation using the DI model in women have never been performed. Taking into account that excitation processes are regulated by anatomically localized structures in the atrial walls and other (see anatomy) structures, we believe that changes in distensibility on the one hand, and in electrolyte balance on the other hand, are reflected in the proteomic composition.

Therefore, the aim of the work was to search for the relationship between proteome changes and some indices of myocardial electrical excitation under conditions of 3-day DI.

## Materials and methods

The experiment with female DI (NAJADA-2020) was conducted at the Institute of Biomedical Problems of the Russian Academy of Sciences from September 7 to 30 November 2020. Six healthy women (age 30.17 ± 5.5 years) participated in the experiment. Each participant had two menstrual cycles (MC) during the study period, including the pre-immersion period and the post-experiment follow-up period. The mean duration of MC was 28.4 ± 2.8 days. In order to unify the influence of hormonal status on the investigated clinical-instrumental and laboratory parameters, the start of baseline studies was timed to the first day of the second cycle. The start of immersion in all participants was on the 7th day of the cycle and its completion was on the 10th day.

The conducted studies were approved by the Bioethical Commission of the Institute of Medical and Biological Problems of the Russian Academy of Sciences (protocol No. 544 of 16 July 2020) and were in full compliance with the principles of the 1964 Helsinki Declaration of Human Rights. Each study participant voluntarily signed an informed consent after the potential risks, tasks, and nature of the upcoming study were explained to her (Pastushkova et al., 2012).

Background examinations were performed on day 5 of the cycle (2 days before immersion), and post-immersion examinations on day 12 (the second day after leaving the immersion). Thus, all blood samples were collected during the follicular phase of the menstrual cycle.

The capillary blood (40 µL) was extracted by puncturing the phalanx of the finger using an automatic scarifier. Sampling was performed with an automatic micropipette with further transfer to filter paper, which was dried and later stored at −20°C. Sample preparation included the following steps: recovery, incubation, trypsin inactivation, deoxycholate precipitation and extraction.

The obtained mixture of tryptic peptides was analyzed by liquid chromatography-mass spectrometry based on a Dionex Ultimate3000 nano HPLC system (Thermo Fisher Scientific, USA) and a timsTOF Pro mass spectrometer (Bruker Daltonics, USA). The mass spectrometric analysis was carried out by parallel accumulation sequential fragmentation (PASEF) data acquisition method. It was measured in the m/z range from 100 to 1700 Th. Ionic mobility were in the range of 0.60–1.60 V - s/cm^2^. Functional annotation of proteins was performed using the String web resource (https://string-db.org) (Pastushkova et al., 2023).

To assess bioelectrical processes in myocardium, an electrocardiogram (ECG) was recorded in the supine position for 5 min in 3 standard leads from the limbs, followed by analysis of microvibrations characterizing electrophysiological properties of myocardium by the method of dispersion mapping (ECG DC) (Ivanov and Sula et al., 2009).

ECG was recorded and analyzed using the hardware-software complex “Cardiovisor” (Russia). To obtain signals of low-amplitude fluctuations of the ECG complex, several consecutive cardiac cycles were synchronized in 30-second segments of a 5-minute recording.

The variance characteristics were calculated for 9 groups (G1-G9), which reflect the degree of severity and localization of electrophysiological disturbances in the atrial and ventricular myocardium during the phases of de- and repolarization (G1 - right atrial depolarization, G2 - left atrial depolarization, G3 - end of right ventricular depolarization, G4 - end of left ventricular depolarization, G5 - right ventricular repolarization, G6 - left ventricular repolarization, G7 - symmetry of ventricular depolarization, G8 - intraventricular blockades, G9 - electrical symmetry of leads).

The total value of all groups of dispersion deviations is the integral “index of microalternations” (IM), which varies from 0% to 100%. The greater its values, the greater the deviations from the norm. Values not exceeding 15% are considered normal. The amplitude of microvariations of the T wave (T-tooth alternation) was also calculated.

To analyze the data set characterizing bioelectrical processes in the myocardium, we used the methods of cluster, discriminant and factor analysis. The *t*-test (*p*-value <0.05) has been used to detect significant protein concentration differences between the experiment points. The whole calculations were performed with the help of STATISTICA 12 statistical program package.

## Results

ECG DC, in our opinion, is one of the most informative techniques used to screen the functional state of the heart muscle during DI. Changes in dispersion characteristics can be detected earlier than in standard ECG analysis.

The variance mapping scores were analyzed using factor analysis. The obtained factor loading pattern revealed the most significant factor in the analyzed sample (describing 65% of the total variance) - “synchrony of myocardial electrical excitation,” which was formed by indicators with the corresponding correlation coefficients: IM (−0.7), T-tooth alternation (−0.7) and G9 (−0.9), reflecting synchrony of depolarization and repolarization in the corresponding heart sections.

All dry blood spot (DBS) samples were analyzed using the PASEF method implemented on timsTOF Pro instruments. Altogether 1256 different proteins were identified in the samples (Pastushkova et al., 2023). Two proteins CAN1_HUMAN and THRB_HUMAN, significantly correlating with ECG dispersion mapping, which reflects electrical processes in the myocardium, were identified when analyzing the protein composition of blood spots of volunteers (Table 1).

**TABLE 1:** ECG DC indices included in the factor “synchronization of myocardial electrical excitation” and their correlations with CAN1_HUMAN (calpain) and THRB_HUMAN (prothrombin) proteins.

Variable	SS Effect	Df Effect	MS Effect	SS Error	dF Error	MS Error	F	p
CAN1_HUMAN	2.492143	1	2.492143	12.9127	28	0.461169	5.403975	0.027566
THRB_HUMAN	4.038610	1	4.038610	24.3395	28	0.869267	4.645995	0.039870

Protein annotation was performed by investigating the physiological processes that are regulated with the participation of calpain and prothrombin (Dewitt and Hallett et al., 2020).

Factor analysis in this study did not reveal a significant effect of 3-day dry immersion on biological processes of atrial repolarization and depolarization.

**FIGURE 1:**
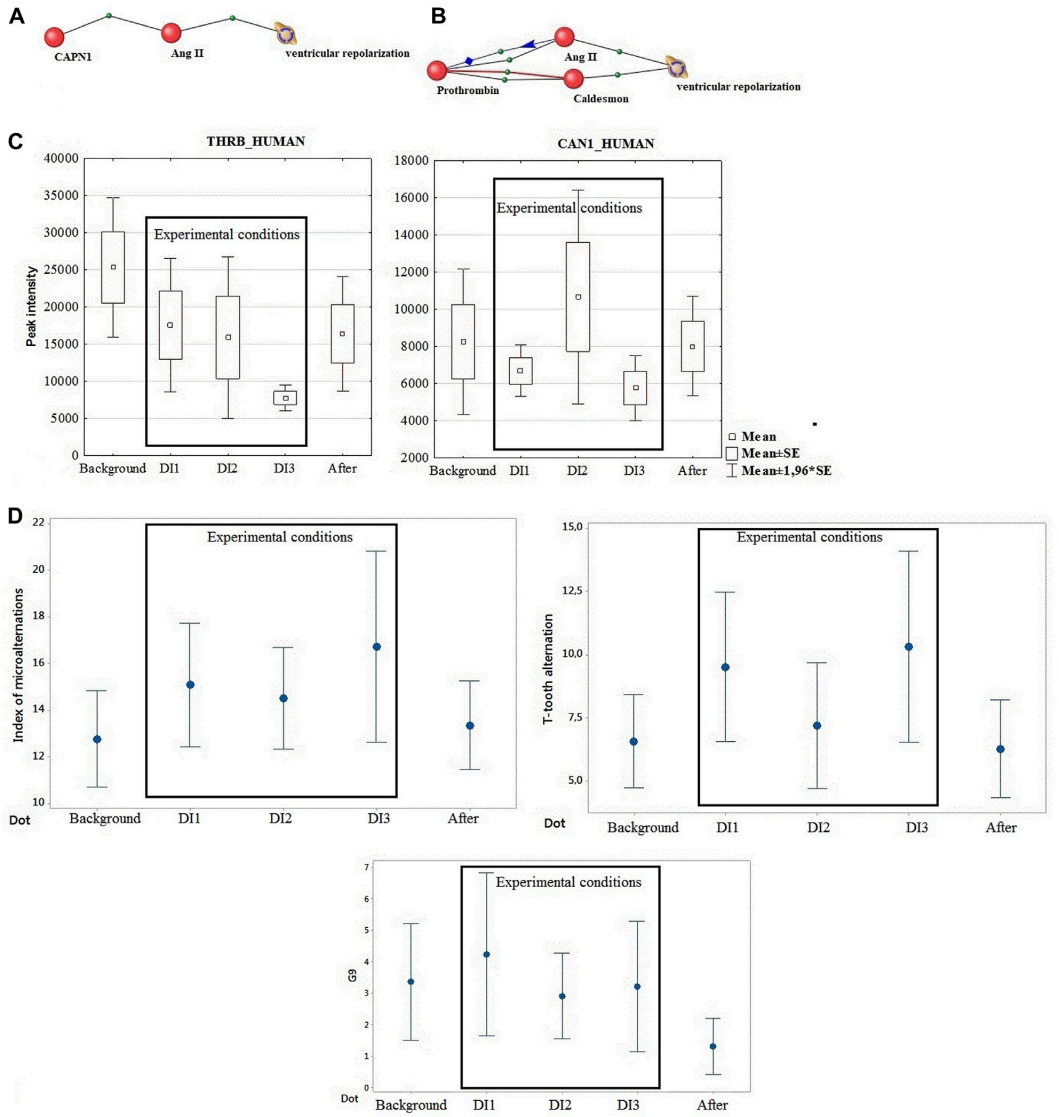
**(A)** Calpain binding via one mediator protein to the process of ventricular repolarization. **(B)** Connection of prothrombin protein through two mediators (angiontensin 2 and caldesmon) with the process of ventricular repolarization Blue line – regulation, Red line-involvement, Black line – association. **(C)** Directionality of calpain and prothrombin dynamics under the influence of DI. **(D)** Dynamics of DC ECG parameters (IM, T-tooth alternation, G9).

For this purpose, an attempt was made to graphically depict the biological relationship between calpain1 and the process of cardiac ventricular repolarization using AndVisio software (Figure 1A) (Ivanisenko et al., 2015). It turned out that calpain 1 through one protein mediator angiotensin 2 regulates the process of ventricular repolarization.

Repolarization is the phase during which the original resting potential of the nerve cell membrane is restored after the passage of a nerve impulse. Ventricular repolarization can be significantly altered by many factors. In the context of this work, it is a change in the mechanoelectrical feedback loop during thoracocranial redistribution of blood and reduction of cardiac output accompanied by myocardial structural rearrangement.

Calpains are known to be a family of cysteine proteases directly activated by Ca2+ and regulated by the endogenous specific inhibitor calpastatin. Among them, calpain-1 and calpain-2 are the most abundant and well-known isoforms (Sorimachi et al., 2011). Calpain-1 - calcium-regulated non-lysosomal thiol-protease which catalyzes limited proteolysis of substrates involved in cytoskeletal remodeling and signal transduction. Overexpression of calpain-1 in cardiomyocytes leads to increased global calpain activity and cardiac remodeling even in the absence of significant changes in intracellular Ca2+ (Galvez et al., 2007). The calpain/calpastatin system is involved in the development of maladaptive hypertrophy triggered by numerous pathologic stimuli (Wang et al., 2018; Aluja et al., 2019) and as indicated in several studies, leads to platelet aggregation, myocardial ischemia, and ultimately heart failure (Zatz and Starling et al., 2005; Wang et al., 2021). It was shown that in ventricular cardiomyocytes of newborn rats angiotensin II increased the expression of calpain 1 (Cao et al., 2022).

Prothrombin, one of the most important factors of the blood coagulation system, which showed a connection with ventricular repolarization, tended to decrease by the end of the experiment. It has been shown that experiments on modeling microgravity conditions do not cause activation of coagulation, although an increase in the peak and rate of thrombin formation was observed after the end of the experiments (Cvirn et al., 2015; Waha et al., 2015). Accordingly, prothrombin may be associated with observable phenotypic and functional changes post DI. Figure 1B shows the relationship of prothrombin protein through the mediator angiontensin 2 and the second mediator caldesmon with the process of ventricular repolarization.

During this study, the dynamics of calpain 1 and prothrombin changes were identical. This fact led us to focus on common mediators for the effects of these two proteins. The significant change in the mediator of both proteins, angiotensin II, is noteworthy (Figures 1A, B). These data are consistent with the changes in angiotensin levels and hemodynamic changes known from literature data under DI conditions of different duration (Pastushkova et al., 2012).

There is increasing evidence that angiotensin II (Ang II) is associated with the occurrence of ventricular arrhythmias. However, little is known about the electrophysiological effects of Ang II on ventricular repolarization. It is known that the fast component of the K(+) delayed rectifier current (I(Kr)) plays a critical role in cardiac repolarization, and Ang II via the protein kinase C pathway-associated AT(1) receptors in ventricular myocytes exerts an inhibitory effect on I(Kr)/hERG currents. This is thought to be a potential mechanism that elevated levels of Ang II are involved in the occurrence of arrhythmias in cardiac hypertrophy and heart failure (Wang et al., 2008).

The second intermediary (Figure 1B) is the actin- and myosin-binding protein Caldesmon (CALD1 gene). This protein is implicated in the regulation of actomyosin interactions in smooth muscle and non-muscle cells. Caldesmon promotes actin binding of tropomyosin, which enhances the stabilization of actin filament structure. It inhibits actomyosin ATPase in muscle tissues by binding to F-actin. The effect is reduced by calcium-calmodulin and increased by tropomyosin. It was shown that saldesmon cooperates with actin, myosin, two molecules of tropomyosin and with calmodulin (Huber et al., 1993).

## Discussion

Disturbance of synchronization of electrical excitation of the heart as a reflection of the state of the circuit of mechano-electrical regulation of the heart under conditions of hemodynamic influence of DI was investigated for the first time. Proteomic regulation of atrial and ventricular depolarization and repolarization processes and ECG DC parameters were evaluated. Factor analysis excluded a reliable influence of DI on biological processes of atrial repolarization and depolarization.

Ventricular repolarization disorders in the population are frequent, asymptomatic, and have multiple causes. Ventricular repolarization disorders are based on congenital individual features of electrophysiological processes in the myocardium, leading to early repolarization of its subepicardial layers, such as: 1. Additional conduction pathways.2. Uneven course of the processes of de- and repolarization of the ventricles. 3. Dysfunction of the autonomic nervous system. 4. Electrolyte disturbances (hypercalcemic theory) (Shulenin et al., 2007).

In DI conditions, in which the state of synchronization of electrical excitation of the heart was assessed, such factors were changes in the structure of cardiac muscle and volume of heart chambers in DI (Westby et al., 2015; Perhonen et al., 2001).

Studying the changes in the cardiovascular system during DI showed the gradual engagement of electrical (an increase in the amplitude of the QRS complex) and then energetometabolic (a decrease in the heart rate and alteration of the water-electrolyte balance) processes in the myocardium; the most pronounced changes were detected on the 5th day of DI (Ivanov et al., 2011). The correlation between changes in hydro-electrolyte balance and its regulation and modifications of electrophysiologic propagation of myocardial excitation, increase in dispersion of intrinsic small oscillations of cardiac potential were described. Changes in hydro-electrolyte balance could cause protein and energy shifts that were reflected, especially, in the rate of ventricular repolarization. The significant (more than 2-fold) growth of the centralization index at the end of SI indicates that the inclusion of the central regulation mechanisms in the processes of adaptation was an appropriate response aimed at compensating the primary changes in the water-electrolyte balance and hemodynamics (Larina et al., 2011). The multi-factor genesis of changes in ventricular repolarization requires more profound studies of proteomic and electrolyte parameters which affect the regulation of the functional state of the heart under simulated and real extreme conditions.

Thus, for the first time the regulation of synchronization of electrical excitation of the heart under conditions of 3 days DI with the participation of women was evaluated at the level of changes in the proteomic composition of blood. Factor analysis in this study excluded a significant effect of dry immersion for 3 days on biological processes of atrial repolarization and depolarization. By bioinformatic analysis of ANDvisio proteomics data from the total list of blood proteins in DI, two proteins related to the biological process of ventricular repolarization through two mediators (angiotensin II and caldesmon) were identified. These data correlate with the results of the DC ECG on changes in ventricular repolarization in all participants during DI. The multifactorial genesis of changes in ventricular repolarization requires further in-depth studies of proteomic and electrolyte circuits of cardiac regulation during DI of different durations to confirm this hypothesis, including gender differences.

## Data Availability

The datasets presented in this study can be found in online repositories. The names of the repository/repositories and accession number(s) can be found below: http://www.proteomexchange.org/, PXD027654.
